# The relations among worry, meta-worry, intolerance of uncertainty and attentional bias for threat in men at high risk for generalized anxiety disorder: a network analysis

**DOI:** 10.1186/s12888-020-02849-w

**Published:** 2020-09-14

**Authors:** Lei Ren, Zhou Yang, Yidi Wang, Long-Biao Cui, Yinchuan Jin, Zhujing Ma, Qintao Zhang, Zhongying Wu, Hua-Ning Wang, Qun Yang

**Affiliations:** 1 Department of Clinical Psychology, School of Medical Psychology, Fourth Military Medical University, Xi’an, China; 2 Department of Economics, University of Washington, Seattle, USA; 3 College of Education, Northern Arizona University, Flagstaff, USA; 4 Department of Psychiatry, Xijing Hospital, Fourth Military Medical University, Xi’an, China

**Keywords:** Generalized anxiety disorder, High risk, Network analysis, Attention bias, Meta-worry, Intolerance of uncertainty, Worry

## Abstract

**Background:**

Improving the psychotherapies for generalized anxiety disorder (GAD) is dependent on a deeper understanding of the relations between GAD and its associated cognitive factors. In the present study, we investigate how the core feature of GAD (i.e., worry) and its associated cognitive factors, such as meta-worry, intolerance of uncertainty, and attention bias towards threat, relate to each other in men at high risk for GAD.

**Methods:**

We used network analysis to explore the relations among these variables in a cross-sectional sample of 122 men at high risk for generalized anxiety disorder. Specifically, we computed the expected influence and predictability of each variable.

**Results:**

In the final network, we found that worry and meta-worry had the highest expected influence and predictability. In contrast, attention bias towards threat showed the lowest expected influence and predictability. The estimates of the expected influence of the nodes were stable (correlation stability coefficient = 0.52).

**Conclusions:**

The present study is the first to investigate the relations among worry, meta-worry, intolerance of uncertainty, and attention bias towards threat in men at high risk for generalized anxiety disorder. These findings indicate that worry and meta-worry may play important roles in the present network. The implications for clinical interventions and future studies are discussed.

## Background

Generalized anxiety disorder (GAD) is characterized by excessive and uncontrollable worry about a series of events or activities and is usually accompanied by other nonspecific psychological and physical symptoms that last at least 6 months [1]. This chronic anxiety disorder is one of the most common mental health problems and some representative epidemiological surveys show that the lifetime prevalence is 4.3–5.9% [2]. Additionally, GAD is particularly prevalent in primary care settings and occurs in 7–8% of patients [3]. Individuals with GAD have considerable role impairment and a high comorbidity with depression [4]. If GAD is not treated promptly, its prognosis is poor [5].

Both pharmacotherapies and psychotherapies have shown efficacy in the treatment of GAD [6, 7]. However, with GAD, clinicians and patients are more likely to consider psychotherapies than pharmacotherapies [2]. Among these psychotherapies, cognitive behavioral therapy (CBT) is often considered a first-line therapy because the evidence for the use of CBT is strongest compared with other psychotherapies [7]. Although CBT can effectively reduce symptoms in as many as 50% patients with GAD, it is unclear how best to treat patients who do not respond to these therapies or who respond only partially [8]. Improving the psychotherapies for GAD is dependent on a deeper understanding of the relations between GAD and its associated cognitive factors [9].

There are several cognitive models that focus on cognitions as key factors driving the development and maintenance of GAD (specifically, the core symptom of GAD: worry) [9]. These models include the intolerance of uncertainty model (IUM) [10, 11], the metacognitive model (MCM) [12], and the cognitive-motivational framework (CMF) [13]. These three cognitive models interpret generalized anxiety from different cognitive perspectives, with relevant empirical supports and theory-based treatment strategies [9].

The IUM emphasizes intolerance of uncertainty (IU) as a crucial factor in the development and maintenance of GAD [14, 15]. Previous studies have found that there is a strong correlation between IU and worry [9, 15]. IU is defined as “a dispositional characteristic that arises from a set of negative beliefs about uncertainty and its connotations and consequences” [16]. IU often triggers a chain reaction of worry, negative problem orientation, and cognitive avoidance [17]. Furthermore, individuals who have high IU are more inclined to treat ambiguous phenomena as unacceptable and threatening, thus causing a negative problem orientation, an inability to take action, and an avoidance response style [18, 19]. Thus, they will be more likely to fall into the process of worry. Under this model, the main goals of GAD therapy are increasing the patient’s tolerance and acceptance of uncertainty [20]. Some randomized clinical trial results with moderate to large effects also support this intervention [21–23].

The MCM proposes negative metacognitive beliefs that consist of the uncontrollability of worry and the dangerousness of worry (e.g., “I can’t control my worries” or “my worries will make me ill”) as a central component in the development and maintenance of GAD [24]. In the MCM, two different types of worry exist in individuals with GAD [24–26]. Type-1 worry is worry about external events and internal noncognitive events. It is a strategic choice to cope with a potentially threatening situation, dependent on the activation of positive meta-beliefs about worrying (e.g., “My worries prompt me to prepare in advance”) [24, 27]. In general, these positive beliefs are normal and not necessarily pathological. In the process of Type-1 worry, negative meta-beliefs about worry can be activated because of social learning experiences, internal emotional regulation, and external sources of information [25]. Individuals with GAD may begin to worry that their Type-1 worries are uncontrollable and dangerous. Such “worry about worry” is designated as meta-worry, or Type-2 worry [9, 25]. A large number of researchers have confirmed that there is a strong correlation between negative meta-beliefs (especially “worries are uncontrollable and dangerous”) and worry frequency or severity [9, 26]. These results have important implications for psychotherapy for individuals with GAD, where the uncontrollability and dangerousness of worries should be regarded as a priority target, as in metacognitive therapy (MCT) [12, 19, 28]. Encouragingly, an increasing number of studies indicate that MCT is an effective therapy for individuals with GAD [27, 29, 30] and that it may be more effective than the current “gold-standard” therapies, pharmacotherapies and CBT [27, 31], as well as other psychotherapies such as intolerance-of-uncertainty therapy (IUT) and applied relaxation (AR) [32, 33].

The CMF indicates that attention bias (AB) towards threat is a key process in the causation and maintenance of anxiety [13, 34]. AB towards threat is considered a steady trait-like characteristic that runs automatically, outside the process of consciousness [13, 35, 36]. By strengthening the worrier’s ability to detect and selectively deal with threat cues, AB can lead to excessive and uncontrollable worries [37]. In addition, individuals with GAD or high worries show a significant AB compared with healthy control samples [38]. Hence, attention bias modification training (ABMT) has been developed to decrease anxiety by applying implicit training programs and hundreds of repeated trials that aim to decrease AB [35, 39]. However, conventional ABMT shows disappointing clinical efficacy in reducing anxiety [13, 34]. The complex relations between AB and anxiety require further investigation to improve ABMT for individuals with GAD or high worries [38].

The network approach is an important and innovative approach for mathematically analyzing and visually displaying the relations among complex variables [40, 41]. It is driven by data and is not dependent on prior assumptions of causality among variables [41, 42]. The network consists of two components: nodes, which stand for objects, and edges, which represent the relations between objects [43]. In light of the research conducted using this approach, mental disorders are believed to arise from the direct interplay between symptoms [44–46]. This approach can also give several centrality and predictability indicators for each node to quantify their importance and controllability in the entire network [47, 48]. Thus, the network approach has become increasingly popular in the field of psychopathology in the past few years because it allows people to explore the complex interplay among the symptoms of mental disorders, provides an alternative way to conceptualize mental disorders, and could have direct implications for more accurate and effective treatment [44, 45, 47]. Recently, an increasing number of studies have shown that adding important and meaningful nonsymptom components such as attention bias towards threat [49], resilience factors [50], emotion regulation [51], reproductive biomarkers [52], and genetic risk scores [53] as nodes in related networks is both empirically feasible and theoretically enriching [47, 54]. Therefore, by relying on the methodological advantages of network analysis (especially the centrality and predictability indicators of each node), we hope to clarify how the core symptom of GAD and its associated cognitive factors relate to each other in men at high risk for GAD, and we hope to quantify the importance and predictability of each variable in the present network, so as to provide some references for related interventions and future research.

Research shows that women and men report significant gender differences in their worry and associated cognitive variables [55, 56]. Theoretical research and psychological care regarding men’s mental health are all in the early stage of development [57, 58]. Therefore, this study focuses on men’s mental health and investigates these variables in individuals at high risk for GAD.

In the present study, we use network analysis to investigate how the core feature of GAD (i.e., worry) and its associated cognitive factors, such as meta-worry, intolerance of uncertainty, and attention bias towards threat relate to each other in men at high risk for generalized anxiety disorder. We are particularly interested in the expected influence and predictability of each variable.

## Methods

### Ethics statement

The independent Ethics Committee of the First Affiliated Hospital of the Fourth Military Medical University approved the implementation of this study (number: KY20182047-F-1). In addition, all participants signed a written informed consent form before participation. They were informed that the present study involved one computer-based task and three scales, and they were ensured that there was no risk of harm in this study and that the results would be kept strictly confidential. As thanks for their participation, we compensated them (approximately 7 US dollars) and taught some simple and practical methods (e.g., breathing relaxation) to ease their anxiety when they experience such feelings.

### Participants

The present study is a cross-sectional study. An initial sample of 1286 men undergraduate students majoring in clinical medicine at the Fourth Military Medical University completed the Generalized Anxiety Disorder 7-Item Questionnaire (GAD-7), a brief and valid scale for screening for GAD and evaluating its severity in clinical practice and research [59]. A total of 127 potential participants with no self-reported diagnosis of any mental illness and sum-scores going beyond the clinical cut-off point (GAD-7 ≥ 10) of Spitzer et al. [59] were preliminarily selected to participate in our study, and we defined these potential participants as people who were at high risk for GAD [60]. Then, we contacted all of them, and 124 individuals expressed their willingness to take part in further investigations. Finally, 122 individuals completed our study (GAD-7: range = 10–21, M = 12.50, SD = 2.74, and internal consistency = 0.72).

### Measures

The Penn State Worry Questionnaire (PSWQ) is a widely used, reliable, and well-validated assessment that measures the degree to which worry (WO) is general, excessive, and uncontrollable in respondents [61, 62]. The PSWQ has 16 items and each item ranges from 1 (“not at all typical of me”) to 5 (“very typical of me”). The internal consistency of these 16 items in the present study was good (α = 0.83).

The Meta-worry Questionnaire (MWQ) consists of 7 items that typically reflect the common danger themes of mental and physical catastrophe because of worry [24]. Moreover, each item has two response scales: one for measuring the frequency of meta-worry and another for measuring the degree to which the respondents believe the meta-worry at the time of meta-worry occurrence. There is a direct association between meta-worry frequency and the presence of GAD, whilst the influence of meta-worry belief on the presence of GAD is mediated by meta-worry frequency [24]. Thus, we used the meta-worry frequency scale to measure meta-worry (MW). The frequency scale is a four-point scale and each item ranges from 1 to 4 (point marked as “never”, “sometimes”, “often”, and “almost always”, respectively). The internal consistency of this scale in the present study was good (α = 0.80).

The 12-item Intolerance of Uncertainty Scale (IUS-12) is a short, efficient, psychometrically sound scale for measuring IU [63, 64]. Items are rated on a five-point Likert scale ranging from 1 (“not at all characteristic of me”) to 5 (“entirely characteristic of me”). The internal consistency of this scale in the present study was good (α = 0.82).

To capture AB among the respondents, we implemented the dot-probe task (DPT), one of the most commonly utilized tasks for this purpose [65–67]. The DPT in the present study was designed by materials provided by Tel Aviv University/National Institute of Mental Health (https://people.socsci.tau.ac.il/mu/anxietytrauma/research.html). The stimuli for the DPT were 20 pictures consisting of 5 men and 5 women (each individual had one neutral face picture and one angry face picture), and the whole process was programmed in E-Prime 2.0.

In the course of each trial, two pictures of the same individual appeared on the screen in pairs (either neutral-neutral face pairs or angry-neutral face pairs). These 55-mm × 55-mm pictures were presented above and below the fixed cross with a 14-mm gap between them. The present DPT consisted of 120 trials in total (40 neutral-neutral and 80 angry-neutral face pairs). Each trial consisted of four steps: (1) a 500-ms fixation, (2) a 500-ms face-pair cue, (3) an arrow with a direction (probe) that appeared in the position of one of the faces and continued until the participants made a response using the left or right button on the mouse, and (4) a 500-ms intertrial interval. The participants were required to press the left or right button on the mouse as quickly and accurately as possible based on the direction of the arrow shown on the screen. Probes had the same probability of appearing on the top or the bottom for neutral or angry face cues and pointing to the right or the left.

We obtained the AB score by comparing the reaction times (RTs) in the trials for the two probe conditions described as follows. In the angry probe condition, the probe appeared in the position of the angry face in the angry-neutral face pairs, whereas in the neutral probe condition, the probe appeared in the position of the neutral face in the angry-neutral face pairs. The AB score was computed as the average RTs for neutral probes–angry probes. A higher positive score represented a faster response speed when the probe replaced the angry face versus the neutral face, indicating a greater AB towards threatening cues [68]. Before calculating the AB score, we dealt with outliers and errors in the DPT of each participant as follows. Trials with incorrect responses and RTs < 150 ms or > 2000 ms were excluded (0.73% of the trials in the neutral probe condition; 1.26% of the trials in the angry probe condition). Then, RTs that were more than 2.5 SDs below or above each participant’s mean for each probe condition were excluded (2.52% of the trials in the neutral probe condition; 2.96% of the trials in the angry probe condition) [69]. The split-half reliability (first-half/second-half split method) with Spearman–Brown correction of the DPT in the present study was poor (*r* = 0.18).

This study process was conducted in the following order: DPT, PSWQ, MWQ, and IU-12.

### Network analysis

Gaussian graphical models (GGMs) were used to fit our data [70, 71]. GGMs are undirected networks in which an edge depicts a partial correlation between two nodes after the influence of all other variables in the dataset has been controlled for [72]. We tested the assumption of normality using the Kolmogorov-Smirnov test. Unfortunately, the significance levels of MW and IU were still *p* < 0.05 after the huge transformation. Therefore, we have taken the suggestion of a reviewer and used nonparametric Spearman rho correlations as input for our analyses (for more related details, see Epskamp and Fried, 2018) [72]. In addition, sparse networks should be preferred because they are easier to interpret and are more stable [42, 50]. The graphical least absolute shrinkage and selection operator (LASSO) was run to regularize the partial correlation network [73]. This process causes small partial correlations to be driven to zero, thereby causing them to not appear in the final graph. Thus, the final network is a parsimonious and sparse network. We used the R package *qgraph* to calculate this network [74]. The *qgraph* package provides an extended Bayesian information criterion (EBIC) to identify the tuning parameter that optimizes the fit and parsimony of the model, and it gives a specific hyperparameter gamma value [75]. We set gamma to 0.5, as suggested by a previous study, which should effectively balance the sensitivity and specificity in selecting true edges [76].

The network was graphed based on Fruchterman-Reingold algorithm (“*spring*” layout from package *qgraph*). This algorithm locates the nodes with stronger correlations near the center of the network, with the nodes with weaker correlations being located near the periphery of the network [77]. The blue edges in the network represent positive partial correlations, whereas the red edges in the network represent negative partial correlations. The thicker the edges are, the greater the partial correlations between two nodes.

Recent studies have shown that strength is the most reliable centrality index, and the centrality indices of betweenness and closeness seem especially unsuitable for assessing the importance of nodes in psychological networks [78, 79]. Node strength is the sum of the absolute value of the edge weights attached to a node, and it may misinterpret the actual effect of nodes on the rest of the network when there are negative edge weights in the network [42, 80]. Thus, we calculated the expected influence (EI). This measure has replaced node strength in the most recent studies due to the evidence that it effectively considers both positive and negative edges within the network [80, 81]. EI was z-scored and computed with the R package *qgraph* [74]. Higher EI values indicate greater importance in the network [51]. We also calculated the predictability of each node by using the R package *mgm* [48]. Predictability is defined as the variance in a node that is explained by all its neighboring nodes (i.e., how the value of a node is determined by all of its neighbors on an interpretable absolute scale). Predictability characterizes the controllability of the network: nodes with high predictability indicate that we can control them through their neighboring nodes in the network while nodes with low predictability indicate that we have to look for other variables out of the network or directly intervene on the node itself [48, 82].

We estimated the robustness of our network by using the R package *bootnet* [83, 84]. First, we evaluated the accuracy of our edge weights by computing 95% confidence intervals (CIs) using a nonparametric bootstrap approach (2000 bootstrap samples). A narrower CI leads to more accurate estimation of edge weights, thereby increasing the accuracy of the centrality index. Second, we estimated the stability of the centrality metrics by computing the correlation stability (CS) coefficient using a case-dropping bootstrap approach. The CS coefficient is the largest number of cases that can be dropped from the entire study to maintain the correlation between the original centrality index and the subsample for at least 0.70 with 95% probability. The value of the CS coefficient should not be below 0.25 and should preferably be higher than 0.50 [83]. Third, bootstrapped difference tests (2000 bootstrap samples and α = 0.05) for the edge weights and node EIs were performed to evaluate whether there is a significant difference between two edge weights or two node EIs.

## Results

The demographic data and descriptive statistics of each variable are shown in Table 1.

**Table 1:** Demographic data and descriptive statistics of each variable

	M	SD	EI	Pre
Age	21.01	1.57		
Level of education	14.83	1.15		
Worry (WO)	59.07	6.99	0.64	0.53
Meta-worry (MW)	13.07	3.26	0.82	0.50
Intolerance of uncertainty (IU)	42.63	5.86	−0.08	0.39
Dot-probe task (DPT)				
Neutral probe condition	502	86		
Angry probe condition	497	92		
Attention bias towards threat (AB)	4.62	15.88	−1.38	0.10

Abbreviations: *M* mean, *SD* standard deviation, *EI* expected influence, *Pre* predictability

**Fig. 1:**
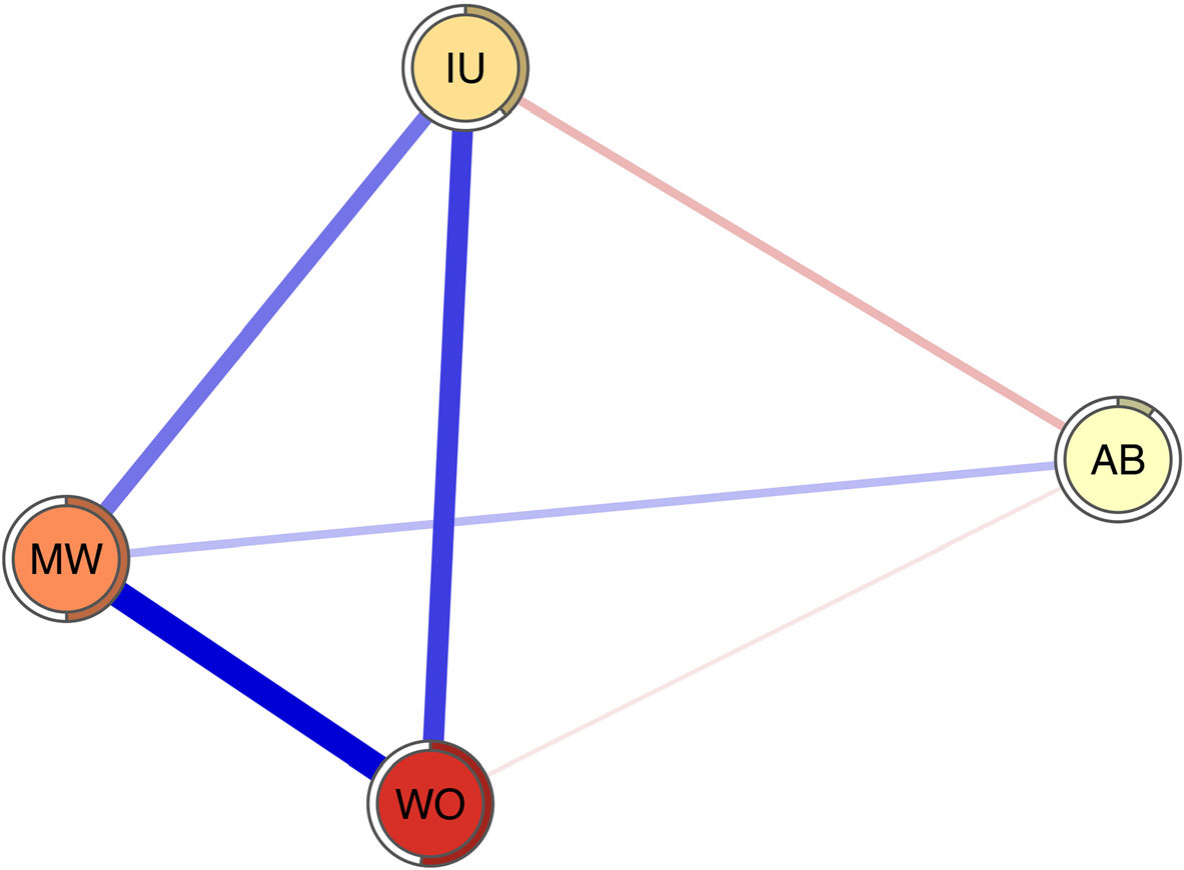
Regularized partial correlation network. Blue edges represent positive correlation, red edges represent negative correlation. The rings around nodes depict its predictability. AB = attention bias towards threat; MW = meta-worry; IU = intolerance of uncertainty; WO = worry

The final network is shown in Fig. 1. An edge in this network represents a partial correlation between two nodes after the influence of all other nodes has been controlled for. Moreover, we regularized the partial correlation network by running the graphical LASSO to obtain a parsimonious and sparse network. Several characteristics were immediately obvious. There was a correlation between each variable and the other variables. The regularized partial correlations between WO and MW, between WO and IU, between MW and IU, between MW and AB, between AB and WO, and between AB and IU were 0.41, 0.31, 0.22, 0.11, − 0.04, and − 0.12, respectively. The predictability of each node was shown as a ring around the node and this ring represented the percentage of the variance in a node explained by all its neighboring nodes. The predictability of WO, MW, IU, and AB was 0.53, 0.50, 0.39 and 0.10 respectively, and the average predictability was 0.38 (see Table 1).

**Fig. 2:**
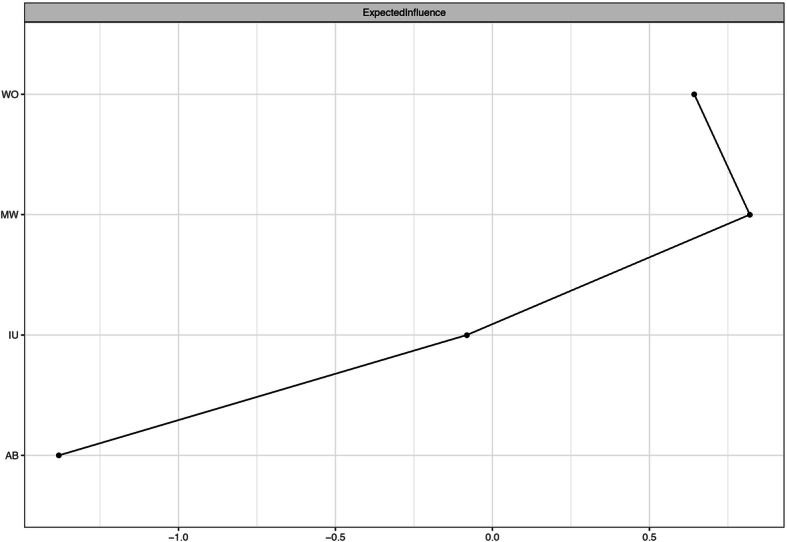
Z-scored expected influence of each variable. AB = attention bias towards threat; MW = meta-worry; IU = intolerance of uncertainty; WO = worry

The z-scored EI values for each variable in our network (see Table 1; Fig. 2) were calculated to assess their relative importance. The two variables having the greatest expected influence value were MW and WO. This result indicated that, from a statistical perspective, they were the most important nodes in the present network. AB exhibited the lowest expected influence value. This result indicated that, from a statistical perspective, AB was the least important node in the present network. The correlation between EI and predictability was 0.99.

**Fig. 3:**
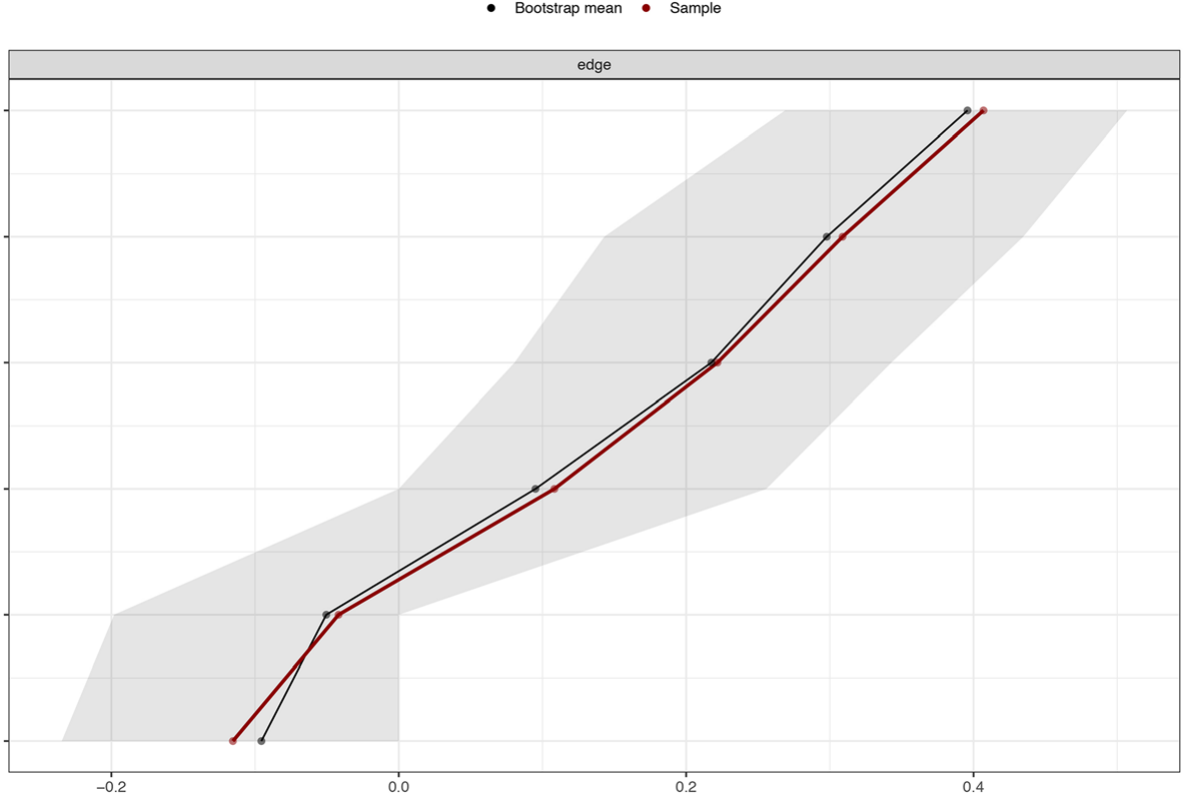
Accuracy of edge weights. The sample edge-weight is depicted by red line and the bootstrap mean edge-weight is depicted by black line. The bootstrapped confidence interval is depicted by gray area

**Fig. 4:**
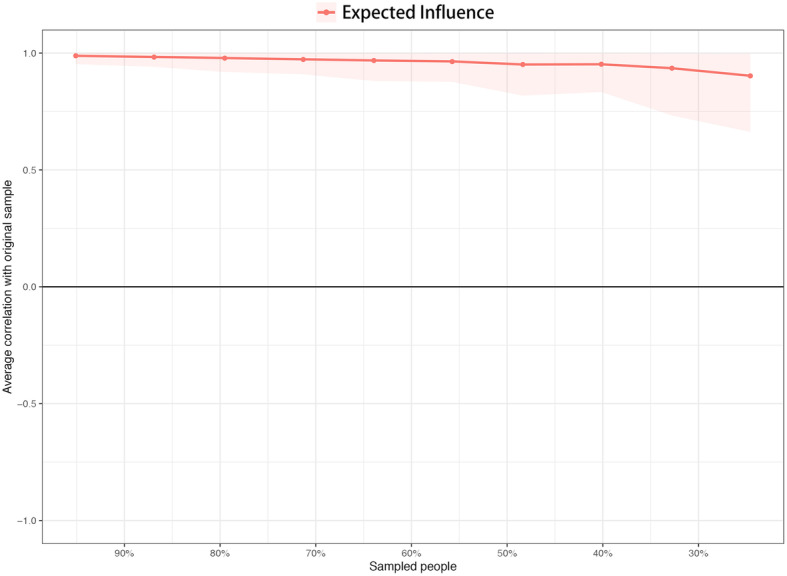
Stability of node expected influences. The red line depicts the average correlation between expected influence in the whole sample and subsample with the red area depicting the 2.5th quantile to the 97.5th quantile

Figure 3 shows the relatively small CIs of the edge weights obtained from the 2000 bootstrap samples. Considering that our network had 122 participants and only 4 nodes, these CIs indicated that the edge weight estimation was accurate. The CS coefficient of EI was 0.52, indicating that the node EI estimation was sufficiently stable (see Fig. 4). The bootstrapped difference tests for the edge weights suggested that the edge weights between WO and MW were significantly different from those between AB and IU, between AB and WO, and between AB and MW; the edge weights between WO and IU were significantly different from those between AB and IU and between AB and WO; and the edge weights between MW and IU were significantly different from those between AB and IU and between AB and WO (see Fig. S1 in Additional file 1). The bootstrapped difference tests for the node EIs suggested that the node EIs of AB were significantly different from those of WO, MW and IU; there are no significant differences among the EIs of WO, MW and IU (see Fig. S2 in Additional file 1).

## Discussion

Our analysis is the first to use a regularized partial correlation network approach to investigate the relations among WO, MW, IU, and AB in men at high risk for generalized anxiety disorder. In this regularized partial correlation network, we identified the network structure and assessed the expected influence and predictability of each variable. Putting the core symptom of GAD and its meaningful cognitive factors in one network could provide novel insights for us to understand the related aspects of psychopathology as well as some references for interventions for men at high risk for GAD [47, 49].

The regularized partial correlations between WO and MW and between WO and IU are the strongest in the present network. These findings are consistent with those of many previous studies that indicate strong associations between worry and worry-relevant cognitive constructs, specifically negative meta-beliefs and IU [9, 19, 85]. These results may also indicate that the MCM and IUM are good theoretical conceptualizations of GAD (specifically, the core symptom of GAD: worry). Moreover, the regularized partial correlation between WO and MW is larger than that between WO and IU even though there is no significant difference between these two edges. This result indicates that the MCM may have a higher ability to conceptualize GAD (specifically, the core symptom of GAD: worry) than the IUM. WO shows a large EI in the present network (only lower than MW), which suggests that targeting WO could lead to general benefits in the rest of the other cognitive factors considered in the network. Notably, there are no significant differences among the EIs of WO, MW and IU, which indicates that there may be no significant differences among the general benefits arising from targeting any one of these three variables. In addition, WO has the highest predictability, which suggests that WO is strongly influenced by its neighboring nodes in the network. This result may provide some important insights for future interventions for worry. For example, this research suggests that we could intervene on WO not only via other related factors that are not included in the network or by intervening on WO itself but also via strong neighboring nodes (MW and IU). In particular, it should be noted that predictability is the upper bound estimation [48]. Previous studies have disclosed bidirectional relationships between worry and IU and MW in which a change in one variable partially explained the change in another variable [24, 86]. Some clinical trials have proven that both MCT and IUT are effective treatments for GAD, and both of them can decrease IU and negative beliefs about worrying [32, 33]. Given that MCT primarily utilizes common CBT interventions and that IUT primarily increases tolerance and acceptance of uncertainty and given the positive results of a previous study and the average predictability of WO, MW and IU in the present study [20, 27], we propose that it is practical and not difficult for cognitive behavioral therapists to integrate CBT, MCT, and IUT in their practice for men. Such integration may lead to a better therapeutic effect. Future research can explore how to effectively integrate these three therapies.

MW has the largest EI in the present network, which suggests that targeting MW could lead to general benefits in the rest of the other variables considered in the network. However, it should be noted that there are no significant differences among the EIs of WO, MW and IU. In addition, MW shows a high predictability (only lower than WO), which suggests that MW is strongly influenced by its neighboring nodes in the network. This may provide some important insight, as it suggests we can control MW not only though other related factors that are not included in the network or intervening on MW itself but also via its strong neighboring nodes (WO and IU). In particular, it should be noted that predictability is within the upper bound estimation [48]. According to MCM, it is very feasible to change MW by intervening on WO [24]. A previous study has shown that IUT can decrease negative beliefs about worrying significantly [32]. It should be noted that the MW in our study only reflects negative metacognitive belief that worry is dangerous [24]. As Wells suggested, a cross-sectional study of the relationship between meta-worry and worry must depend on measurement of the meta-worry danger instead of uncontrollability domain when using PSWQ to capture the generality, excessiveness, and uncontrollability domains of worry [24, 61, 62].

IU shows a lower EI when compared with WO and MW. It should be noted that there are no significant differences among the EIs of WO, MW and IU. A significant body of research has proven that IU plays an important role in the development and maintenance of WO [15, 87]. This body of research is bolstered by the fact that IUT has achieved positive clinical effects in treating WO in individuals with GAD [32]. However, more studies are needed to determine the relative importance of IU in GAD symptoms and associated cognitive factors.

AB has the only EI that differs significantly (and is significantly lower) from the other nodes in the network, which suggests that targeting AB could lead to few benefits in the rest of the other variables considered in the network. In fact, AB keeps directly related to all other variables even though these connections are slight. This result indicates that the CMF may have a limited ability to conceptualize GAD (specifically, the core symptom of GAD: worry). On the one hand, this may be due to method variance, where the regularized procedure shrinks the smallest edges to zero. On the other hand, it may be because all the variables with the exception of the AB measure are self-report measures, which are likely to show stronger relations. In addition, AB shows a low predictability, which suggests that AB is slightly influenced by its neighboring nodes in the network. It should be noted that the reliability of related results and conclusions regarding AB are greatly reduced due to the poor split-half reliability of the DPT in the present study. Moreover, an increasing number of studies show that the reliability of the DPT is poor, which means that the DPT may not be suitable for assessing AB [88, 89]. Therefore, more fine-grained and reliable AB estimates such as eye-tracking, event-related potentials and functional magnetic resonance imaging, can be used in future studies [13, 90–93]. Nonetheless, we tested whether participants exhibited AB towards threat significantly greater than 0, and the result, t_(121)_ = 3.417, *p* < 0.001, means that there is a significant difference. However, it remains hard to explain these data without healthy and clinical control groups.

There are several limitations in the present study worth mentioning. First, all the participants were male undergraduate students majoring in clinical medicine, which limits the universality of our conclusions. Medical students may be more sensitive to MW because of their professional knowledge and they are more aware of the dangers. Moreover, as mentioned above, research shows that women and men report significant gender differences in their worry and associated cognitive variables [55, 56]. The resulting network structure and related indicators (such as the expected influence and predictability of each node) could differ when tested in women. Therefore, the discussion of the findings and the derived potential implications of the present study should be limited to men. Second, this study sampled only individuals at high risk for GAD, rather than clinical samples. It is urgently necessary to verify our results in clinical samples. Third, as suggested by a reviewer, expanding the network to include the full set of GAD symptoms in the GAD-7 as nodes might indeed provide a more integrative view of the relative contribution of different cognitive factors to worry and other GAD symptom levels as well as the relations among the different symptoms. However, due to the current sample size and the heterogeneity of the symptom measures, we did not include the full set of GAD symptoms as nodes in the present network. Future studies can further explore such networks. Fourth, the relationship discussed here cannot be considered causal in our cross-sectional study, and longitudinal data are needed. Recently, experience sampling methodology (ESM) has become increasingly popular in the field of mental health because of its ability to capture variables over time [94, 95]. Future studies can use ESM to explore the time causality of these variables and obtain personalized network models for personalized intervention. Finally, the reliability of the related results and conclusions regarding AB is greatly reduced due to the poor split-half reliability of the DPT in the present study. More fine-grained and reliable AB estimates, such as eye-tracking, event-related potentials and functional magnetic resonance imaging, can be used in future studies [13, 90–93].

## Conclusions

The present study is the first to use network analysis to investigate the relations among worry, meta-worry, intolerance of uncertainty, and attention bias towards threat in men at high risk for generalized anxiety disorder. The results indicate that the largest partial correlation is between worry and meta-worry and that worry and meta-worry have the highest expected influence and predictability in the present work. These findings indicate that worry and meta-worry may play important roles in the present network. The implications for clinical interventions and future studies are discussed.

## Supplementary information

**Additional file 1 **: **Figure S1.** Bootstrapped difference test for edge weights. **Figure S2.** Bootstrapped difference test for node expected influences.

## Data Availability

The datasets used and/or analysed during the current study are available from the corresponding author on reasonable request.

## Abbreviations

GAD: Generalized anxiety disorder
CBT: Cognitive behavioral therapy
IUM: Intolerance of uncertainty model
MCM: Metacognitive model
CMF: Cognitive-motivational framework
IU: Intolerance of uncertainty
MW: Meta-worry
MCT: Metacognitive therapy
IUT: Intolerance-of-uncertainty therapy
AR: Applied relaxation
AB: Attention bias towards threat
ABMT: Attention-bias modification training
GAD-7: Generalized anxiety disorder 7-item Questionnaire
PSWQ: Penn state worry questionnaire
WO: Worry
MWQ: Meta-worry questionnaire
IUS-12: 12-item Intolerance of uncertainty scale
DPT: Dot-probe task
RT: Reaction times
GGM: Gaussian graphical models
LASSO: least absolute shrinkage and selection operator
EBIC: Extended Bayesian information criterion
EI: Expected influence
CIs: Confidence intervals
CS: Correlation stability
M: Mean
SD: Standard deviation
Pre: Predictability
ESM: Experience sampling methodology
