# Supplementary material for: The relations among worry, meta-worry, intolerance of uncertainty and attentional bias for threat in men at high risk for generalized anxiety disorder: a network analysis
Source: BMC Psychiatry. 2020 Sep 14;20:452. doi: 10.1186/s12888-020-02849-w (PMC7491186; doi:10.1186/s12888-020-02849-w)
Supplement: Supplementary file 1 — Additional file 1 : Figure S1. Bootstrapped difference test for edge weights. Figure S2. Bootstrapped difference test for node expected influences. [file 12888_2020_2849_MOESM1_ESM.docx]

**Additional file 1**

**Bootstrapped difference test for edge weights and node expected influences**

1. Figure S1. Bootstrapped difference test for edge weights
2. Figure S2. Bootstrapped difference test for node expected influences


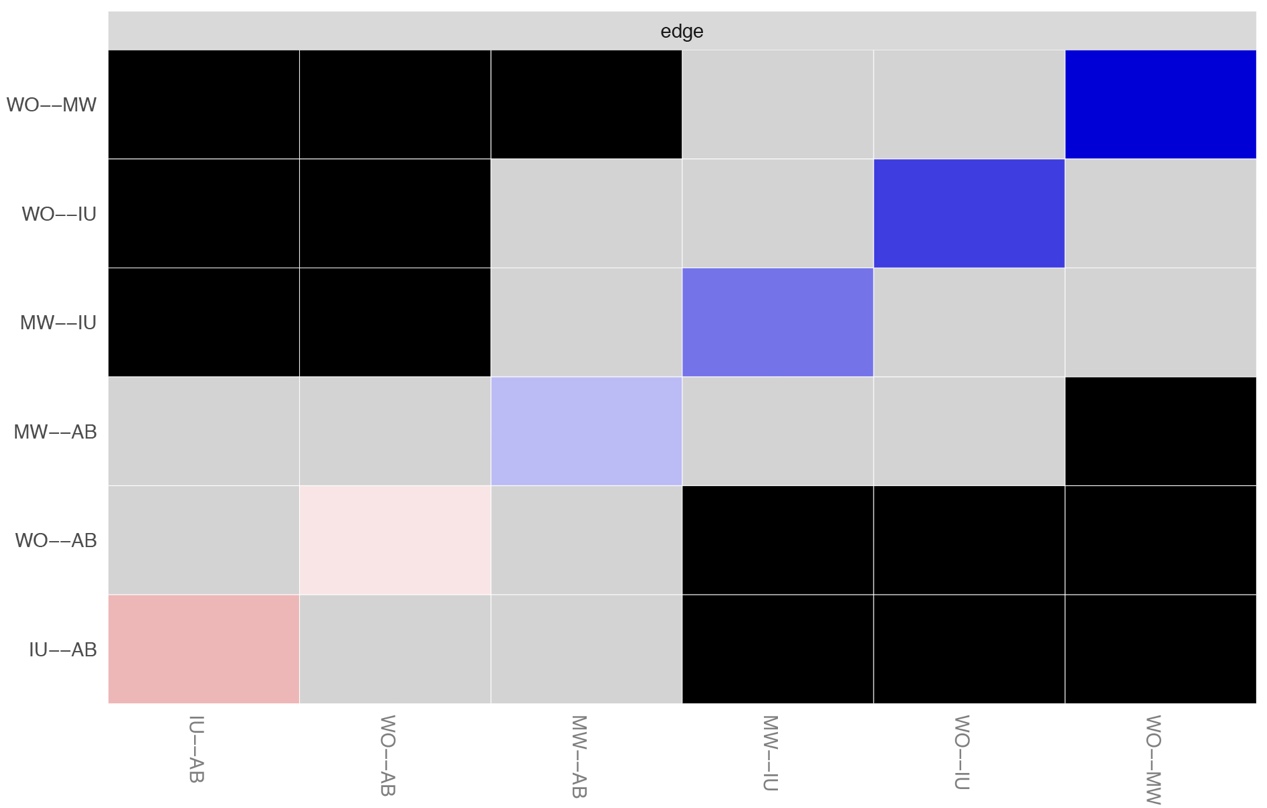


Fig. S1. Bootstrapped difference test for edge weights.

*Note*: Gray boxes indicate edge weights that do not differ significantly from one another, while black boxes indicate edge weights that do differ significantly. Blue and red boxes on the diagonal correspond to edge weights with positive and negative correlations, respectively.


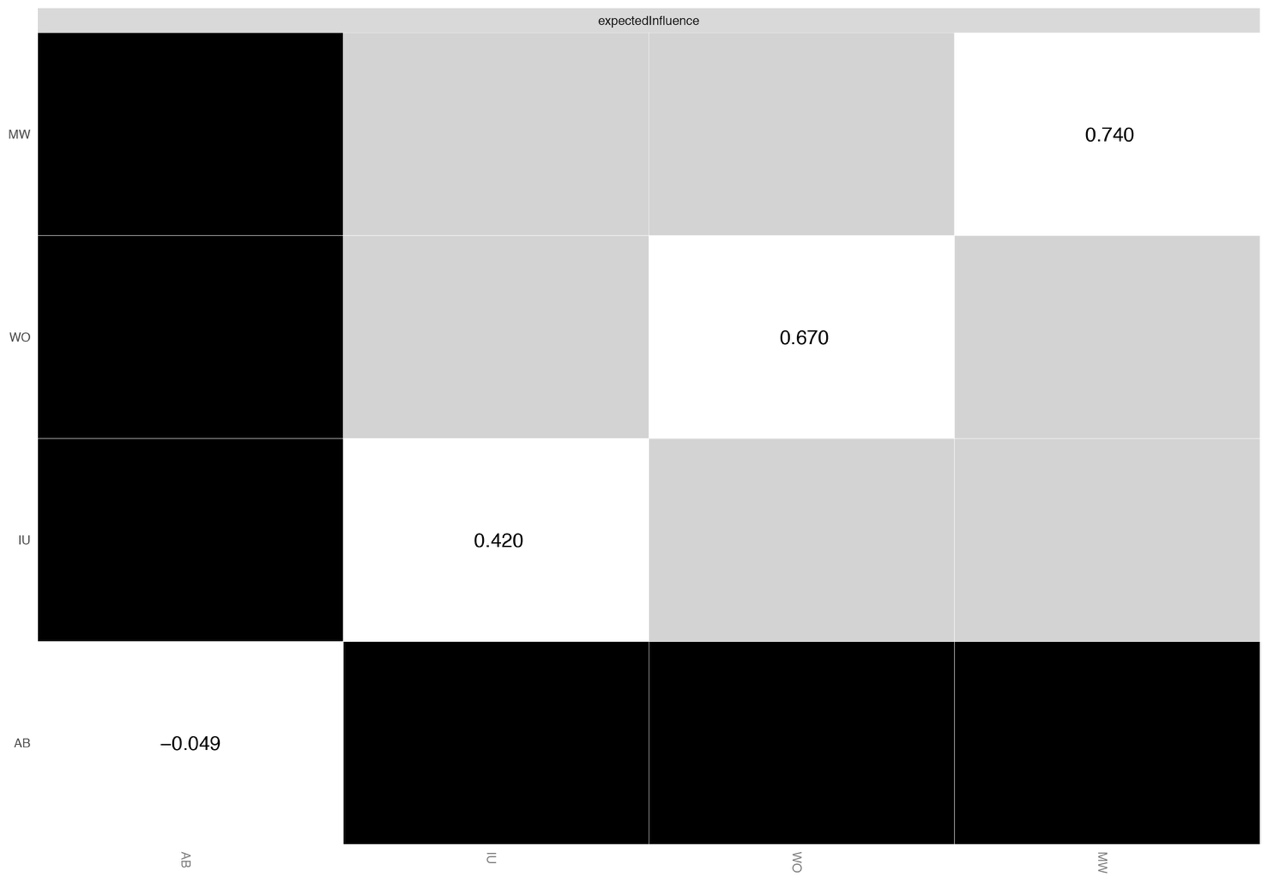


Fig. S2. Bootstrapped difference test for node expected influences.

*Note*: Gray boxes indicate node expected influences that do not differ significantly from one another, while black boxes indicate node expected influences that do differ significantly. The number in the white boxes (i.e., diagonal line) represent the value of node expected influences.
